# Feasibility and safety of same-day discharge after paediatric vagus nerve stimulator implantation for medically intractable epilepsy: a retrospective safety analysis

**DOI:** 10.1007/s00381-026-07425-3

**Published:** 2026-08-18

**Authors:** Joshua Pepper, Richard Walsh, William B. Lo

**Affiliations:** 1 Complex Epilepsy Surgery Service, Birmingham Children’s Hospital, Birmingham, UK; 2 Department of Neurosurgery, Birmingham Children’s Hospital, Birmingham, UK

**Keywords:** Epilepsy, VNS, Vagal nerve stimulator, Day case surgery

## Abstract

**Objective:**

Vagal nerve stimulation (VNS) is an established adjunctive treatment for children with medically intractable epilepsy. It is common practice to admit these children overnight for observation due to concern of airway compromise and other medical complications. We wanted to determine the safety of vagal nerve stimulator insertion in the immediate peri-operative period to determine if same-day discharge is feasible and safe in children.

**Methods:**

We conducted a retrospective review of all paediatric patients (age less than 19 years of age) who underwent vagal nerve stimulator (VNS) implantation at our institution between January 2002 and December 2022.

**Results:**

In total, 197 patients were included in the analysis. Mean age at surgery was 11.1 years (SD 4.1). Median length of stay was 1 day (IQR 1–1); 21 children (10.7%) were discharged the same day. There were no intraoperative complications. No child (0%) had an immediate postoperative surgical complication. Three children had postoperative medical complications (1.5%), all of which occurred in the first hour after surgery. Developmental delay was present in 95% of documented cases.

**Conclusions:**

These findings support the safety of same-day discharge in appropriately selected patients, with structured criteria and parental guidance.

## Introduction

Medically, refractory epilepsy in the paediatric population presents significant treatment challenges. Vagal nerve stimulation (VNS) has been demonstrated as an effective adjunctive therapy, reducing seizure frequency and improving quality of life in select patients [1–4]. The surgical procedure involves careful dissection in the neck region to identify and wrap the stimulator lead around the vagus nerve, situated posterior to the common carotid artery and jugular vein [5].

Postoperative management in our unit traditionally includes an overnight stay to allow 24 h of inpatient observation to monitor for both surgical and medical complications in our cohort of patients who often have complex medical needs and communication difficulties. The most feared complication is that of a postoperative cervical haematoma causing airway compromise requiring immediate return to theatre and surgical evacuation which has been reported in under 1% in large series [6, 7]. This practice is particularly cautious in children with developmental delays and learning difficulties, who may be at increased risk of airway compromise and less able to communicate symptoms.

However, the incidence of significant postoperative complications following VNS implantation appears to be low [7, 8]. With increasing emphasis on healthcare resource optimisation and patient-centred care, there is growing interest in safely transitioning selected paediatric patients to same-day discharge protocols after VNS implantation. Although some centres note they discharge patients on the same day [6, 9] as far as we are aware there is no paediatric specific safety data on this and many larger series report keeping patients in for 24 h [7, 10, 11].

This retrospective study aims to review our institutional experience with VNS implantation in a paediatric cohort to assess the risk of immediate postoperative complications with a view to assess the safety and viability of moving to a same-day discharge model.

## Methods

We conducted a retrospective review of all paediatric patients (age less than 19 years of age) who underwent vagal nerve stimulator (VNS) implantation at our institution between January 2002 and December 2022 for medically intractable epilepsy. Patient records, including case notes, operative reports and digital medical records, were reviewed to extract relevant clinical and peri-operative data. Due to the historical nature of some records, data were unavailable for 8 cases, which were excluded from the analysis. The primary aim of this study was to assess the rates of intraoperative and immediate postoperative complications with our intention of assessing the safety of moving to a same-day discharge model. For the purposes of this study, a length of stay (LOS) of 0 days was defined as same-day discharge, while LOS values of 1, 2 and more days represented one, two or more overnight inpatient stays following surgery, respectively.

The patient cohort included our paediatric cohort with medically intractable epilepsy who were selected for VNS implantation based on multidisciplinary assessment.

### Surgical technique

After general anaesthesia, the child is placed supine on the operating table with an interscapular roll, neck extension and head turn to the right. Following a transverse cervical incision and dissection medial to the sternocleidomastoid muscle, the carotid sheath is identified. The carotid sheath is opened anteriorly, and the vagal nerve is identified posterior to and between the jugular vein and the common carotid artery. Approximately 2–3 cm of vagal nerve is dissected free and a vascular loop placed around it. Next, an approximately 5-cm infraclavicular transverse incision is made the length of which is dependent on the pulse generator inserted. A subcutaneous and occasionally a subpectoral pocket is created. The lead is then tunnelled from the cervical incision to the pectoral pocket and passed through. The electrode and anchoring coils are then placed around the vagal nerve and tension-relieving loops secured with the anchors provided. The lead is then connected to the battery and impedance checked. If satisfactory the pulse generator is secured and the wounds are closed in layers and skin closed with subcuticular suture.

### Postoperative management

Postoperative management includes a 24-h inpatient stay to monitor for complications such as haematoma formation and airway compromise. Clinical parameters monitored postoperatively included neurological status, airway patency, mobility, cognition and tolerance of oral intake.

### Immediate postoperative complications

For the purposes of this study, we focused on the timing and nature of intraoperative and immediate postoperative complications. Immediate postoperative complications included all those that occurred either within 4 h from surgery, prior to discharge or in the first 48 h after surgery if the child was still an inpatient or it was reported after discharge.

We classified complications into surgical or medical.

Surgical complications were defined as complications directly attributable to the operative site, surgical dissection or hardware placement and included local neck/wound complications (cervical haematoma with airway compromise, wound breakdown, skin erosion, etc.), local neural/structural issues (recurrent laryngeal nerve injury, vagus nerve injury, dysphagia, vocal cord paresis, etc.), device/hardware related (lead malposition, generator pocket complications, high lead impedance, etc.) and acute postoperative surgical events (bleeding requiring re-operation, acute airway compromise, expanding wound swelling, etc.).

Medical complications were defined as complications related to patient physiology, anaesthesia or events not related to the surgical act itself. This could include cardiovascular (hypotension, bradycardia or arrhythmia, syncope, etc.), neurological (increased seizure frequency, postoperative confusion/drowsiness, etc.), respiratory (anaesthesia related hypoventilation, other events not related to haematoma, etc.) and any other postoperative medical issues (nausea/vomiting, pain requiring unplanned admission, biochemical/haematological abnormalities requiring admission, etc.).

Readiness for discharge was assessed based on return to baseline mobility, cognition and ability to tolerate oral intake the day after surgery.

### Statistical analysis

Statistical analysis was descriptive, using measures of central tendency including means and medians where appropriate, to summarise demographic and clinical variables. Due to the observational and retrospective design, no inferential statistical tests were performed.

## Results

**Fig. 1:**
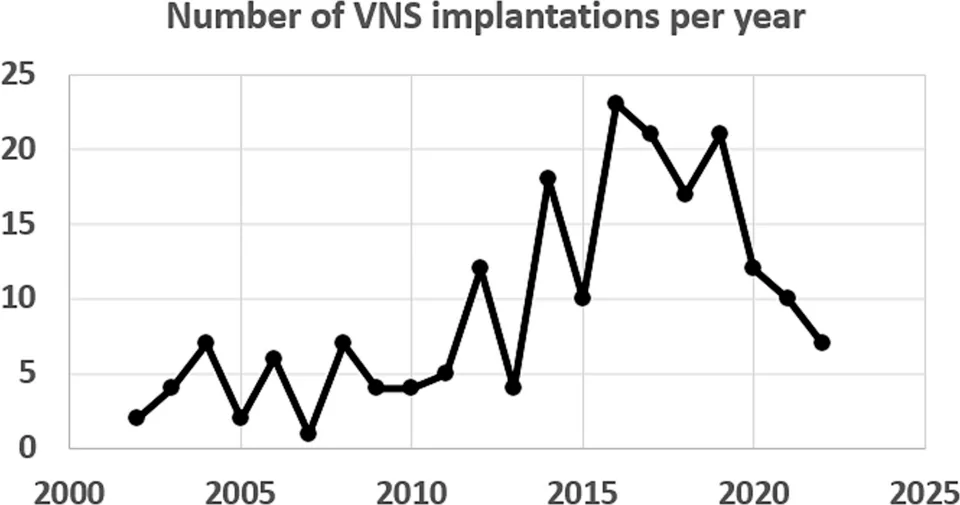
Number of VNS implantations per year

From September 2002 until November 2022, we identified 205 children who underwent primary insertion of a vagal nerve stimulator. Of these, information could not be obtained in 8 children due to the historical nature of some records and due to a change in patient record system at our institution leaving 197 patients in the final analysis. See Fig. 1 for number of surgeries by year.

Mean age at surgery was 11.1 years (SD 4.1 years). Median age was 11.1 years (3.3–19.0 years).

Operations took place on a Monday (*n* = 20), Tuesday (*n* = 13), Wednesday (*n* = 143), Thursday (*n* = 1), Friday (*n* = 6), Saturday (*n* = 1) and Sunday (*n* = 13).

Information on developmental delay was noted in 159 children. Of these, 151 (95.0%) were reported as having developmental delay, 8 (5.0%) as having normal development without learning difficulties.

The underlying cause of the epilepsy if noted can be seen in Table 1.

**Table 1:** Underlying aetiology of epilepsy

Category	*N*
Genetic epilepsy (gene/chromosome/named genetic syndrome)	39
Dravet syndrome	7
Lennox–Gastaut syndrome	25
West syndrome/infantile spasms	6
Myoclonic absence epilepsy	6
Doose (myoclonic–astatic) syndrome	3
Structural malformation (developmental)	5
Perinatal brain injury	6
Infectious/inflammatory (post-encephalitis/meningitis)	9
Metabolic/mitochondrial disorder	2
Generalised epilepsy (unspecified aetiology)	41
Focal epilepsy (unspecified aetiology)	9
Focal epilepsy (multifocal; unspecified aetiology)	4
“Medically intractable” epilepsy (type not specified)	35

The generator model placed in the primary surgery was a NCP M101 (*n* = 6), Pulse M102 (*n* = 11), Demipulse M103 (*n* = 65), AspireHC M105 (*n* = 2), SenTiva M1000 (*n* = 31) and Aspire SR M106 (*n* = 82). Median length of stay (LOS) was 1 day. LOS was 0 days (*n* = 21), 1 day (*n* = 165), 2 days (*n* = 10) and 4 days (*n* = 1). Ten out of 11 children who stayed more than 1 day did so for optimisation of anti-epileptic medications. One out of 11 childen stayed for more than 1 day as they had low systolic blood pressure postoperatively and was kept in for monitoring.

See Table 2 for the number of postoperative surgical and medical concerns in the first 4 h and the first 48 h.

**Table 2:** Surgical and medical complications after vagus nerve stimulator surgery

Timeline	Surgical complications	Medical complications
Intraoperative	0/197 (0%)	0/197 (0%)
First 4 h	0/197 (0%)	3/197 (1.5%)
Between 4 and 48 h after surgery	0/197 (0%)	0/197 (0%)
Return to theatre in the first 48 h	0/197(0%)	0/197 (0%)

### Intraoperative complications

There were no reports of intraoperative medical, anaesthetic or surgical complications.

### Postoperative surgical complications

There were no surgical complications in the first 4 h or the first 48 h after surgery. This gives a surgical complication rate of 0% (0/197).

### Postoperative medical complications

Medical complications occurred in 1.5% of cases (3/197), all presenting within the first 4 h after surgery. All of these children were admitted until the underlying medical issue had resolved. One child, who was an 11-year-old boy at the time of implantation, has a background of epileptic encephalopathy, non-verbal, severe global developmental delay, autism and learning difficulties. Immediately postoperatively, the systolic blood pressure was borderline low so the decision was made to keep the child in hospital for observation. He was given a bolus of intravenous fluids appropriate to their weight and observed for 48 h. He was discharged with no other issues at this time point. A second child with a background of tuberous sclerosis had multiple convulsive seizures starting 1 h after surgery and was observed for 48 h under the supervision of the epileptologist prior to discharge. No surgical complications were noted. The third child had a background of extensive cystic encephalomalacia with cerebral palsy, epileptic encephalopathy, severe developmental delay and was fed via a gastrostomy. They were unable to be weaned from nasally delivered oxygen in the immediate postoperative period. There was no concern for a neck haematoma or other surgical complications. They required suctioning and oxygen overnight and were discharged at baseline the next day.

### Return to theatre

No child had a return to theatre within the first 48 h after surgery. This gives a return to theatre rate of 0%.

## Discussion

Our institutional experience of 197 paediatric VNS implantations demonstrates a negligible incidence of immediate complications within the initial hours after surgery. This suggests that with appropriate patient selection and peri-operative monitoring, same-day discharge may be a safe and feasible alternative to the traditional 24-h inpatient observation period. Prior to discharge, all children are assessed to be at baseline cognition, mobility and oral intake tolerance. Importantly, there were no surgical complications and all medical complications presented within the first hour after surgery suggesting that in appropriately selected patients a same-day discharge model is safe.

The primary concern after VNS implantation is haematoma formation in the neck region, which can precipitate airway obstruction. Early postoperative vigilance is critical to timely identification and management of such complications. In our cohort, no cases of airway compromise, peri-tracheal haematoma or any other issues requiring urgent intervention were identified in this timeframe, consistent with prior literature reporting low complication rates [6–10].

Children with developmental delays and learning difficulties often pose challenges for postoperative assessment due to limited communication abilities and potential comorbidities affecting airway protection. Despite this, our experience suggests that careful neurological and clinical evaluation can reliably establish readiness for discharge. Criteria such as return to baseline cognition and mobility, and tolerance of oral intake, provide practical and objective measures of patient stability.

Transitioning to a same-day discharge model in neurosurgery [12, 13] has potential benefits, including reduced healthcare costs [14], decreased hospital-acquired complications and patient satisfaction [15, 16] so long as comprehensive pre-operative parental counselling and education is given. However, this must be balanced with ensuring patient safety and adequate postoperative support at home.

A recent audit of the National Surgical Quality Improvement Program-Pediatric database [17] in the USA demonstrated that of 3070 children undergoing VNS insertion, 63 had an unplanned readmission within 7 days of the procedure. There was no difference in rates of readmission for same-day discharge versus those with inpatient admissions. While the scope of our study looked at more granular detail in terms of immediate postoperative nursing observations and intraoperative findings, the conclusion is very similar in that moving to a same-day discharge model for vagal nerve stimulator insertion in paediatrics appears to be a safe option.

### Recommended protocol

We recommend that patients should undergo a minimum 4-h observation period and demonstrate return to baseline cognition, mobility, airway status and oral intake prior to discharge. They should have adequate pain control with simple analgesia and families should receive clear postoperative instructions with access to rapid medical review if concerns arise.

Children with significant peri-operative concerns, ongoing oxygen requirements, haemodynamic instability or failure to return to neurological baseline are not suitable candidates for same-day discharge.

### Limitations

A key limitation of the present study is that, despite demonstrating a very low incidence of immediate postoperative complications, it was not designed to identify patient-level predictors of adverse events. Only three medical complications occurred within the cohort, preventing meaningful risk-factor analysis. Consequently, larger multicentre studies will be required to develop and validate evidence-based risk stratification criteria for same-day discharge following paediatric VNS implantation.

Further prospective studies with larger cohorts are warranted to validate these findings and to formalise discharge protocols, including patient and caregiver education, and streamlined pathways for prompt readmission if needed.

## Conclusion

Our review supports the safety and feasibility of a same-day discharge protocol following paediatric VNS implantation for medically intractable epilepsy. Importantly, all complications occurred within the first hour after surgery suggesting a minimum observation period of 4 h represents a safe observation threshold. With stringent discharge criteria and early postoperative monitoring, hospitalisation beyond four hours may be unnecessary in select patients, optimising care delivery without compromising safety.

## Data Availability

No datasets were generated or analysed during the current study.
